# Rural-Urban Disparities in Colorectal Cancer Screening, Diagnosis, Treatment, and Survivorship Care: A Systematic Review and Meta-Analysis

**DOI:** 10.1093/oncolo/oyad347

**Published:** 2024-01-19

**Authors:** Aryana Sepassi, Meng Li, Jason A. Zell, Alexandre Chan, Ila M Saunders, Dana B Mukamel

**Affiliations:** Department of Clinical Pharmacy Practice, University of California, Irvine School of Pharmacy & Pharmaceutical Sciences, Irvine, CA, USA; Department of Health Services Research, University of Texas MD Anderson Cancer Center, Houston, TX, USA; Division of Hematology/Oncology, University of California, Irvine School of Medicine, Irvine, CA, USA; Department of Clinical Pharmacy Practice, University of California, Irvine School of Pharmacy & Pharmaceutical Sciences, Irvine, CA, USA; Division of Clinical Pharmacy, University of California, San Diego Skaggs School of Pharmacy & Pharmaceutical Sciences, La Jolla, CA, USA; Department of Medicine, University of California, Irvine, CA, USA

**Keywords:** colorectal cancer, cancer screening, cancer diagnosis, cancer treatment, cancer survivorship care

## Abstract

**Background:**

Rural residents have a higher prevalence of colorectal cancer (CRC) mortality compared to urban individuals. Policies have been aimed at improving access to CRC screening to reduce these outcomes. However, little attention has been paid to other determinants of CRC-related outcomes, such as stage at diagnosis, treatment, or survivorship care. The main objective of this analysis was to evaluate literature describing differences in CRC screening, stage at diagnosis, treatment, and survivorship care between rural and urban individuals.

**Materials and Methods:**

We conducted a systematic review of electronic databases using a combination of MeSH and free-text search terms related to CRC screening, stage at diagnosis, treatment, survivorship care, and rurality. We identified 921 studies, of which 39 were included. We assessed methodological quality using the ROBINS-E tool and summarized findings descriptively. A meta-analysis was performed of studies evaluating CRC screening using a random-effects model.

**Results:**

Seventeen studies reported disparities between urban and rural populations in CRC screening, 12 on treatment disparities, and 8 on staging disparities. We found that rural individuals were significantly less likely to report any type of screening at any time period (pooled odds ratio = 0.81, 95% CI, 0.76-0.86). Results were inconclusive for disparities in staging at diagnosis and treatment. One study reported a lower likelihood of use of CRC survivorship care for rural individuals compared to urban individuals.

**Conclusion:**

There remains an urgent need to evaluate and address CRC disparities in rural areas. Investigators should focus future work on assessing the quality of staging at diagnosis, treatment, and survivorship care in rural areas.

## Teaser

This article evaluates the literature on differences in colorectal cancer screening, stage at diagnosis, treatment, and survivorship care of patients in rural areas compared with those in urban areas.

**Implications for Practice**

Despite the significant implementation of a policy aimed toward the expansion of the colorectal cancer care continuum to individuals living in rural areas, our results suggest that significant barriers to access to care still exist. Our findings highlight the need for a more in-depth policy and practice approach aimed at reducing disparities in screening, diagnosis, and treatment for rural individuals. Healthcare providers must remain cognizant of these substantial barriers to care and of the limitations in assessing literature evaluating colorectal cancer care disparities for rural populations.

## Introduction

Approximately 14% of the US population lives in a rural area.^1^ Individuals living in rural areas are often subject to poorer health outcomes compared to their urban counterparts. For example, the age-adjusted death rate in rural areas is 7% higher than that of urban areas.^2^ These excess deaths have been linked to a higher prevalence of chronic conditions such as heart disease, chronic lower respiratory disease, and cancer.^3^ This gap in health outcomes and their underlying causes between rural and urban populations reflects a lack of equitable access and use of health services over time.^2-8^ Moreover, less than 8% of all physicians and surgeons in the US practice in rural settings and 64% of rural medical staff report difficulties in finding specialists for patient referrals.^9,10^

A higher incidence of cancer-related cases and mortality are among the most impactful causes of excess deaths among rural populations as a result of these health disparities.^11,12^ While national cancer incidence has declined over time, the incidence and mortality rates of certain cancers have persisted in rural areas, such as those for colorectal cancer (CRC).^13^ In 2016, the annual age-adjusted death rate due to CRC in nonmetropolitan areas was estimated at 17.1 deaths per 100 000 persons, higher than the estimated rate in metropolitan areas (14.0 deaths per 100 000 persons).^13^ This trend was also noted for incident CRC cases, where the age-adjusted incident CRC rate among nonmetropolitan residents was higher than that of metropolitan residents (43.9 cases per 100 000 persons vs 39.6 cases per 100 000 persons, respectively).^13^ Of the hypothesized driving factors behind these outcome disparities in incidence and mortality, those involving the patient’s provider and their care along the CRC care continuum may be particularly impactful.^14^

Patients in rural areas experience limited access to medical and oncology providers and higher-quality care, which may lead to later stages of any cancer diagnosis and a lower likelihood of receiving standard-of-care treatment with supportive care.^12,15^ Therefore, inequities in each of these stages (CRC screening, stage at diagnosis, treatment, and survivorship care) of patients with CRC care may contribute to worsened downstream outcomes. Limited access to CRC screening among rural individuals has been previously characterized.^16^ A systematic review from Wang et al found that the most frequently reported barriers for rural individuals to CRC screening were high cost, lack of insurance coverage, and lack of perceived need for CRC screening.^16^ However, it is unknown to what extent these barriers in screening, in addition to barriers in treatment, stage at diagnosis, or survivorship care differ from urban individuals. To better guide policymakers, decision makers, and healthcare providers in addressing the impact of these hypothesized CRC disparities on cancer-related incidence and mortality for rural patients, it is essential to understand how each of these categories independently contributes to patient with CRC care and subsequent outcomes. Moreover, it is critical to understand how various existing definitions of rural-urban status can affect analyses evaluating CRC care disparities, as different rural–urban categorizations can lead to a variation in results for studies evaluating disparities or barriers in access to care.^17^ To better understand these urban-rural CRC disparities, we performed a systematic literature review to evaluate original research investigating differences in CRC screening, stage at diagnosis, CRC treatment, and survivorship care between rural and urban adults in the US. Secondary to this, we performed a meta-analysis to synthesize findings across these gaps where sufficient data were available.

## Materials and Methods

We conducted a systematic review to assess differences in CRC screening, stage at CRC diagnosis, treatment, and survivorship care between rural and urban adults in the US in accordance with the Preferred Reporting Items for Systematic Reviews and Meta-Analyses Protocols (PRISMA-P).^18^ This study was exempt from IRB approval and informed consent as it collected and synthesized nonidentifiable data from previously published studies. The protocol for this study was approved and made available on PROSPERO (ID #CRD42022350943).

### Data Sources, Search Strategy, and Inclusion/Exclusion Criteria

The review was conducted using PubMed and EMBASE for primary sources of evidence published between January 2012 and July 2022. From our original search, we identified and excluded duplicate indices and screened abstracts for inclusion criteria (Supplementary Materials). Those qualifying for inclusion were further screened using the full-text source and once again screened for final inclusion/exclusion criteria. We used a “snowball” approach in reviewing studies to ensure that references of included studies that also qualified for inclusion were not missed in the original search strategies. We used search terms related to rural populations, colorectal cancer, screening/prevention, treatment, diagnosis, and survivorship care and combined several database-specific search terms, such as Medical Subject Heading (MeSH) terms and free-text search terms. The full search strategy may be found in the Supplementary Materials. We specified eligibility criteria using the PICOT framework (population, intervention, comparator, outcome, time frame), summarized in the Supplementary Materials. We included randomized controlled trials, observational cohort studies, and case-control studies in English evaluating rural vs urban individuals as either a primary or secondary outcome in any of the 4 “disparity categories” (screening, stage at diagnosis, treatment, and survivorship care). We excluded publications that were other systematic reviews or meta-analyses, guidelines, letters to the editor, case studies, ethnographic or qualitative studies, and surveys. We did not exclude studies based on a specific type of definition or categorization method used to distinguish rural–urban individuals nor on any specific age group. We included studies that reported incidence rates for rural and urban populations and studies that only reported regression output(s) as a method of assessment. CRC screenings of interest included fecal-occult blood testing (FOBT), fecal immunochemical testing (FIT), flexible sigmoidoscopies, colonoscopies, CT colonographies, and barium enema exams. Studies evaluating disparities in stage at diagnosis must have reported data on the type of staging definition used (SEER staging classification system, American Joint Committee on Cancer 7th Edition Staging, etc.). Those assessing treatment disparities must have reported data on the type of treatments evaluated. Treatments of interest included surgical resection (laparoscopic colectomy, open resection), chemotherapy, and radiotherapy. For studies that also included data on other types of cancers, we extracted results for CRC only.

### Screening, Study Design, and Data Abstraction

The original search, screening, and abstraction were performed using Covidence (Melbourne, AUS). Three investigators (A.S., M.N., L.C.) screened initial database search results (title and abstract) for studies adherent to the predetermined PICOT criteria. Indices screened and included were screened once more using the publication full text to ensure final study inclusion. Data were abstracted from each full-text publication and included the following: first author, year of publication, study design, geographic location, years of data evaluated, age group, inclusion criteria, rural-urban classification and categorizations, data source, screening types evaluated, staging definition and categories, treatment types evaluated, and results of primary/secondary outcomes. To assess for study bias, 2 investigators (A.S., M.N.) independently reviewed each full-text article using the ROBINS-E tool.^19^ At each stage, any disagreements between reviewers were resolved by discussion, and, if necessary, adjudicated by a fourth reviewer.

### Meta-Analysis of CRC Screening

Data for all outcomes except CRC screening were not sufficient to permit meta-analysis. Therefore, we limited the meta-analysis to studies reporting differences in CRC screening. The primary outcome was the odds ratio (OR) comparing rural and urban populations for any screening method reported at any time. All studies were weighted based on the generic inverse-variance method.^20^ A random-effects model was developed and built on the assumption that between-study variance results from factors other than measured treatment differences.^21^ The random-effects model assumes a normal distribution of between-study variance, which is facilitated by the generally large sample sizes (>100) of the included studies.^21^ All analyses were performed using the “meta” package in R with a significance level of 0.05.^22^ The code for this analysis is publicly available (https://tinyurl.com/3kknzasx).

### Assessment of Heterogeneity and Publication Bias

Quantifiable heterogeneity between studies reporting CRC screening differences was evaluated using the *I*^2^ statistic. The *I*^2^ statistic developed by Higgins et al describes the percent of total variation across studies attributable to heterogeneity beyond random chance.^20^ Generally, values of 0% indicate no heterogeneity, and 25%, 50%, and 75% indicate low, moderate, or high heterogeneity, respectively.^20^ We assumed an acceptable *I*^2^ value of 50% or less.^23^ In the event of an *I*^2^ value > 50%, we specified a priori methods on outlier assessment and removal. We used methods from Viechtbauer and Cheung to identify and remove outlier studies with effect sizes outside of the 95% CI of the original pooled result.^24^ Publication bias was evaluated using Peter’s test and funnel plots, which evaluate the relationship between the effect size of each study and its precision.^25^ If there is a detected systematic relationship between effect size and precision, publication bias may be present. Pooled results were reported for without outliers and with outliers as a pooled odds ratio with 95% CI in a forest plot.

## Results

### Study Characteristics

**Figure 1.**
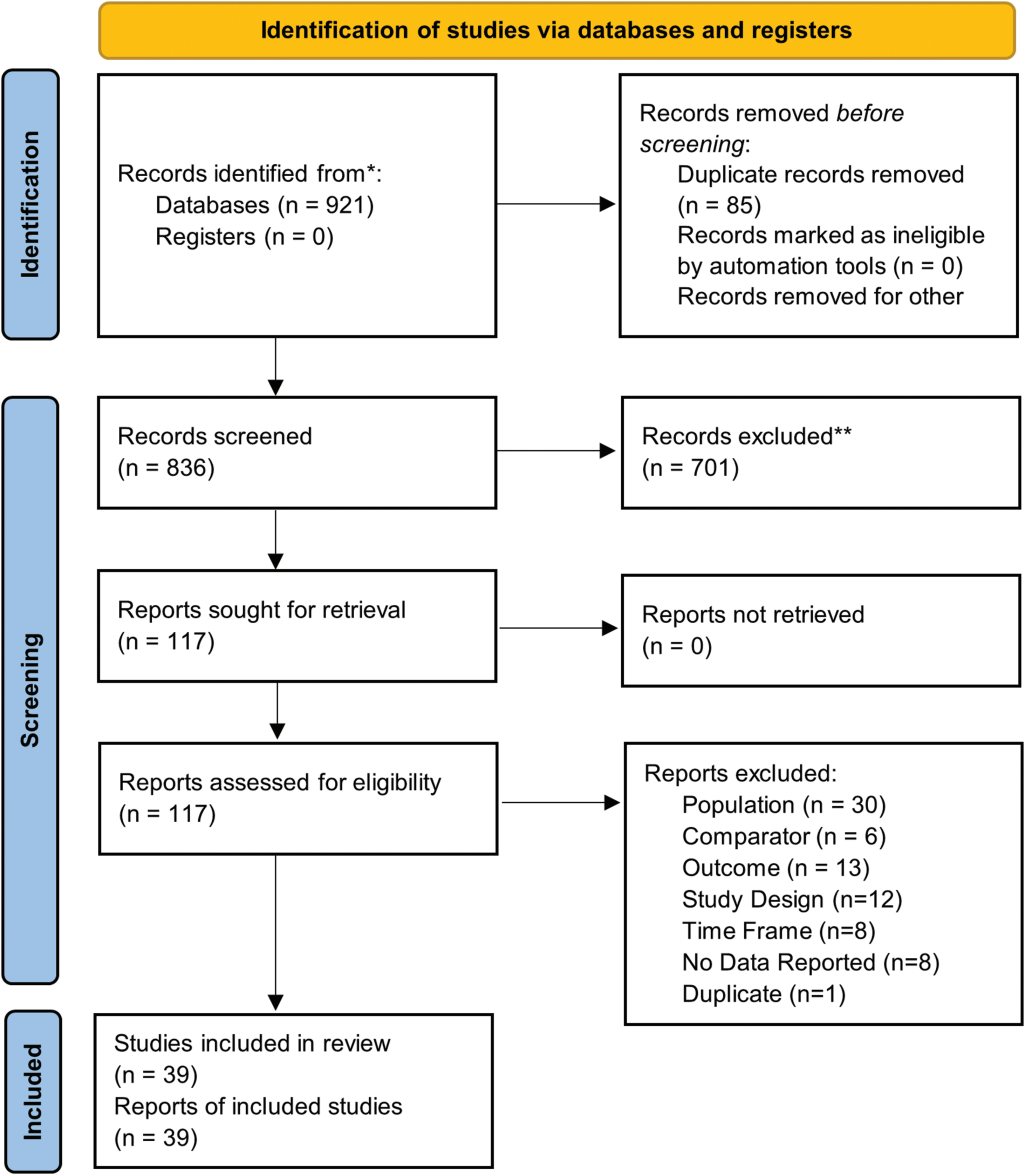
PRISMA flow diagram for screening article selection and evaluation.

A total of 921 studies were initially collected. After screening for inclusion/exclusion criteria, 39 studies published between 2005 and 2022 were included in the final analysis, representing a total of 8 186 449 individuals (6 731 362 urban, 1 455 07 rural, Fig. 1).^26-63^ All included studies were observational.^26-63^ Seventeen studies reported results on screening disparities, followed by 12 reporting treatment disparities, 8 reporting results on staging/diagnosis disparities, and one reporting survivorship care disparities (Table 1).^26-63^ Sixteen studies used national or nationally representative data to perform analyses.^28,31,33,39,41,42,47,49-52,55-59^ The remaining 23 studies reported results for individual states or groups of states ( Supplementary Fig. S1).^26,27,29,30,32,34-38,40,43-46,48,53,54,60-63^ Of these, 7 studies reported disparity data in the Southeastern US, 4 in the Southwest, 6 in the West, 7 in the Midwest, and 4 in the Northeast.^26,27,29,30,32-38,40,43-46,48,53,54,60-63^ Rural-urban definitions varied widely across studies. The majority of studies (15) classified rural-urban individuals using Rural-Urban Commuting Area (RUCA) codes.^30,32,33,35,37,40,43-46,53,59,62,63^ Nine studies used Rural-Urban Continuum Codes (RUCC) for classification.^31,34,39,47-49,51,55,61^ Three studies used Urban Influence Codes (UIC), 2 studies used ZIP codes, and 3 studies used 2000 US Census definitions for classification.^26-29,31,38,50^ Two studies used the Office of Management and Budget (OMB) criteria, 1 study used CDC rural-urban definitions, and 2 studies used Metropolitan Statistical Area (MSA) classifications to define rural-urban individuals.^31,36,41,42,60^ One study used the Medical Service Study Area (MSSA) classification scheme to categorize individuals and later used RUCA codes as a sensitivity analysis.^53^ One study also tested different rural-urban classification criteria (OMB, RUCC, UIC, National Center for Health Statistics definition) as a primary analysis.^31^ Four studies did not explicitly report how individuals were classified into rural-urban categories.^52,56-58^ Most studies evaluated individuals 50-75 years old (23.1%), in line with CRC screening recommendations from the US Preventive Task Force.^28,34-40,42,64^ Risk of bias assessments revealed a mostly overall high risk of bias for included studies (Supplementary Figs. S2, S3). This was primarily driven by a high risk of bias due to missing data, exposure measurement, and confounding.

**Table 1.** Study population age, rural-urban classification, urban, and rural categorization.

Author (Year)	Population age (years)	Year(s) evaluated	Inclusion criteria	Rural-Urban classification	Urban categorization	Rural categorization
Schumacher (2008)	≥50	2004-2007	American Indian or Alaska Native, not pregnant, no active cancer treatment	2000 U.S. Census	Communities with a population of 50 000 or more	Communities with a population of less than 50 000
Ko (2005)	≥65	2000	Medicare beneficiary	ZIP Codes	ZIP codes not coded as rural	All ZIP codes closest to a rural hospital, defined by WA Department of Health
Bennett (2011)	50-75	2008	NR	UIC	UIC Code 1,2	UIC Codes 3-12
Wang (2017)	≥40	2004-2011	NR	Zip Codes	NR	NR
Mojica (2020)	≥50	2013-2015	Newly eligible for Medicaid	RUCA	50 000 or greater density	49 999 or fewer density
Hirko (2022)	≥50	2008-2013	NR	OMB National Center for Health Statistics RUCC UIC	OMB: Population <49 999 NCHS: Codes 1-4 RUCC: Codes 1-3 UIC: Codes 1-2	OMB: Population >49 999 NCHS: Codes 5-6 RUCC: Codes 4-9 UIC: Codes 3-12
Lai (2015)	≥65	2008-2010	Medicare beneficiary	RUCA	NR	NR
Fan (2012)	≥65	2005	Medicare beneficiary	RUCA	NR	NR
Shete (2021)	50-75	2017-2020	Female only	RUCC	RUCC Code 1-3	RUCC Code 4-9
Anderson (2013)	50-75	2010	Reported familial CRC history	RUCA	NR	NR
Ojinnaka (2015)	50-75	2012	NR	MSA	Center City of MSA, outside Center City and Inside County with CC, suburban County	Rural/Non-MSA
Kurani (2020)	50-75	2016-2017	NR	RUCA	NR	NR
Hughes (2015)	50-75	2014	NR	2000 US Census, FAR Definition	“Metropolitan” according to U.S. Census	Counties with populations of up to 25 000 people and at least 45 minutes away from areas of at least 25 000 people (FAR level 2)
Moss (2019)	50-75	2011-2017	NR	RUCC	RUCC Code 1-3	RUCC Code 4-9
Haakenstad (2019)	50-75	2009-2012	NR	RUCA	RUCA Code 0-9	RUCA Code 10 or Higher
Moreno (2020)	18-64	2017	Individuals with a known address	MSA	NR	NR
Carmichael (2020)	50-75	2016	NR	CDC Rural-Urban Definition	Levels 1-4: Large Central Metropolitan, large Fringe Metropolitan, medium Metropolitan, small Metropolitan	Levels 5-6: Micropolitan, non-Core
Alyabsi (2020)	50-64	2013-2015	No diagnosed CRC	RUCA	NR	NR
Hines (2014)	45-85	2000-2007	First incidence of CRC, single tumor, known tumor stage, race not African American or White	RUCA	RUCA Code 1 Suburban: RUCA Code 2-6	RUCA Code 7-10
Leech (2022)	18-64	2010-2015	Not Medicare or Medicaid covered	RUCA	RUCA Code: 1.0, 1.1, 2.0, 2.1, 3.0, 4.1, 5.1, 7.1, 8.1, 10.1	RUCA Code: 4.0, 5.0, 6.0, 7.0, 7.2, 8.0, 8.2, 9.0, 10.0, 10.2, 10.3
Lin (2017)	≥18	2001-2012	NR	RUCA	RUCA Code: 1.0, 1.1, 2.0, 2.1, 3.0, 4.1, 5.1, 7.1, 8.1	RUCA Code: 4.0, 4.2, 5.0, 5.2, 6.0, 6.1, 7.0, 7.2, 7.3, 7.4, 8.0, 8.2, 8.3, 8.4, 9.0, 9.1, 9.2, 10.0, 10.2, 10.3, 10.4, 10.5, 10.6
Zahnd (2018)	NR	2009-2013	NR	RUCC	RUCC Code 1-3	RUCC Code 4-9
Risser (2012)	NR	2004-2008	NR	RUCC	NR	NR
Paquette (2007)	≥20	2000-2003	Primary diagnosis of CRC	RUCC	RUCC Code 1-3	RUCC Code 7, 9
Andrilla (2020)	≥50	2010-2014	Individuals in exclusively urban counties	UIC	UIC Code: 1- 8	UIC Code: 9,10,11,12
Zahnd (2018)	≥1	2009-2013	NR	RUCC	RUCC Code 1-3	RUCC Code 4-9
Al Nasser (2014)	≥18	2009	NR	NR	NR	
Parikh-Patel (2021)	≥18	2004-2014	NR	MSSA	Any area not considered “rural”	Population density <250 people/square mile and no census-defined place with a population >50 000 in the area
Stewart (2013)	≥18	2004-2006	Rectal cancers only, no patients with in situ cancer	RUCA	NR	NR
Panchal (2016)	≥66	2004-2009	Medicare Beneficiary	RUCC	Population of 20 000 or more	Population of <2500
Monson (2014)	NR	2006-2011	Documented surgical resection	NR	NR	NR
Moghadamyeghaneh (2015)	≥18	2009-2012	NR	NR	NR	NR
Patel (2022)	≥18	2008-2017	NR	NR	NR	NR
Chioreso (2019)	≥65	2007-2011	Rectal cancer only	RUCA	NR	NR
Gruber (2015)	≥19	2008-2011	NR	OMB	NR	NR
Short (2016)	≥65	2006-2008	Medicare Beneficiary	RUCC	NR	NR
Hao (2011)	≥15	2000-2004	Non-Hispanic White or Non-Hispanic Black individuals only	RUCA	RUCA Code 1-6 (2-6 as “suburban”)	RUCA Code 7-10
McDougall (2018)	30-75	2004-2012	NA	RUCA	NR	NR

^**^Did not report definitions.

Abbreviations: FAR, frontier and remote; MSA, Metropolitan Statistical Area Codes; MSSA, medical service study area; NR: none reported; RUCA, rural-urban commuting area code; RUCC, rural-urban continuum code; UIC, urban influence code.

### Studies Reporting on CRC Screening Disparities

A total of 1 060 857 individuals were evaluated across 18 studies reporting differences in CRC screening (764 340 urban, 296 517 rural).^27,28,30-33,35-39,41,43,44^ Of the 18 studies reporting differences in CRC screening among rural-urban individuals, the majority included data on colonoscopies (17, 94.4%, Table 2), followed by flexible sigmoidoscopy (15, 83.3%) and FOBT (11, 66.1%).^27,28,30-33,35-39,41,43,44^ Less commonly studied screening methods included FIT, CT colonography, barium enema testing, multitarget stool DNA testing, and “any type of screening.”^28,31,33,38,40,42^ Overall, urban populations consistently demonstrated higher CRC screening rates compared to rural populations across all studies, except for one.^26-43^

**Table 2.** Colorectal cancer screening study characteristics.

Author (year)	Setting	Years evaluated	Data source	Screening types evaluated (time period evaluated)	Urban population (*n*, %)	Urban population screened (*n*, %)	Rural population (*n*, %)	Rural population screened (*n*, %)
Schumacher (2008)	State (AK, AZ, NM)	2004-2007	EARTH survey	Colonoscopy (last 5 years), Flexible sigmoidoscopy (last 5 years)	357 (13.5%)	180 (50.4%)	2 288	410 (17.9%)
Ko (2005)	State (WA)	2000	Physician/Supplier Medicare Part B Analytic File	FOBT, Flexible sigmoidoscopy, Colonoscopy, Barium enema	328 920 (81.8%)	11 610 (3.53%)	73 041 (18.2%)	2 381 (3.26%)
Bennett (2011)	National	2008	BRFSS	“Any screening”	219 (70.9%)	103 (46.9%)	90 (29.1%)	39 (43.0%)
Wang (2017)	State (CA)	2009	CHIS	Flexible sigmoidoscopy, Colonoscopy	30 992 (80.4%)	18 835 (80.4%)	7 513 (19.5%)	4 486 (19.6%)
Mojica (2020)	State (OR)	2013-2015	Medicaid Enrollment & Claims Data	FOBT^***^, FIT, Colonoscopy, Sigmoidoscopy	5183 (57.4%)	920 (62%)	3849 (42.6%)	573 (38%)
Hirko (2022)	National	2008-2013	BRFSS	Flexible sigmoidoscopy, Colonoscopy	1164 (37.1%)	681 (59.0%)	1971 (62.9%)	1078 (54.7%)
Lai (2015)	State (KS)	2008-2010	State cancer registry data linked to Medicare Enrollment Files	FOBT, FIT, Flexible sigmoidoscopy, Colonoscopy, CT colonography, Barium enema	673 (42.4%)	137 (47.0%)	915 (57.6%)	157 (58.0%)
Fan (2012)	National	2005	Medicare Current Beneficiary Survey	FOBT (last 2 years), Colonoscopy (last 5 years), Flexible sigmoidoscopy (last 5 years)	8792^*^ (76.0%)	4880^*^ (55.5%)	3118^*^ (24.0%)	1489^*^ (47.8%)
Shete (2021)	State (PA, KY, OH, UT, VA, MN, AL, OR, KS, WA)	2017-2020	Survey developed using NCI Population Health Assessment	FOBT (last year), Colonoscopy (last year), Flexible sigmoidoscopy (last year)	1749 (60.4%)	1429 (82.0%)	1090 (37.6%)	848 (78.0%)
Anderson (2013)	State (UT)	2010	BRFSS	FOBT (last year), Flexible sigmoidoscopy (last 5 years), Colonoscopy (last 10 years)	3082 (72.3%)	2151 (68.3%)	1178 (27.7%)	693 (56.8%)
Ojinnaka (2015)	State (TX)	2012	BRFSS	FOBT (last 5 years), Colonoscopy (last 10 years), Sigmoidoscopy (last 3 years)	3614 (86.0%)	2602 (72.0%)	589 (14.0%)	404 (68.6%)
Kurani (2020)	State (MN, IA, WI)	2016-2017	EHR Data	FOBT (last year), Multitarget stool DNA test (last 3 years), Flexible sigmoidoscopy (last 5 years), CT colonography (last 5 years), Colonoscopy (last 10 years)	51 100 (35.1%)	NR	94 450 (64.9%)	NR
Hughes (2015)	State (NE)	2014	Survey developed using Health Behavioral Model	FOBT (last year), Sigmoidoscopy (last 5 years), FOBT (last 3 years), Colonoscopy (last 10 years)	193 (49.1%)	170 (88.1%)	200 (50.9%)	151 (75.5%)
Moss (2019)	National	2011-2017	HINTS	“Any screening”	15 315 (86.3%)	10 643 (69.5%)	2421 (13.7%)	1593 (65.8%)
Haakenstad (2019)	State (ME)	2009-2012	Maine Health Data Organization All Payer Claims Database (Medicare & Commercial Claims)	Colonoscopy	284 675 (79.5%)	17 080 (6.0%)	73 344 (20.5%)	3667 (5.0%)
Moreno (2020)	National	2017	IBM Watson MarketScan Commercial Claims and Encounters Database	CT colonography	NR^**^	NR	NR	NR
Carmichael (2020)	National	2016	BRFSS	FOBT (last year), Colonoscopy (last 10 years), Sigmoidoscopy (last 5 years),	NR	68.0%	NR	66.0%
Alyabsi (2020)	State (NE)	2015	BlueCross BlueShield of Nebraska Claims Data	FOBT (last year), Colonoscopy (last year)	28,312	FOBT: 10758 (38.0%) Colonoscopy: 14 213 (50.2%)	30 460	FOBT: 18885 (62.0%) Colonoscopy: 15230 (50.0%)

^*^Results reported for “time appropriate screening” across all screening types.

^**^Authors reported results in person-years. Urban person-years: 14 721 852. Rural person-years: 1 746 419. Screening CT colonography, urban: 2.67 per 100 000 PYs. Diagnostic CT colonography, urban: 5.58 per 100 000 PYs. Screening CT colonography, rural: 1.04 per 100 000 PYs. Diagnostic CT colonography, rural: 4.14 per 100 000 PYs.

^***^All evaluated within the year after study individuals turned 50 years old.

Abbreviations: BRFSS, Behavioral Risk Factor Surveillance Survey; CHIS, California Health Interview Survey; EARTH, Education and Research Toward Health; EHR, Electronic Health Record; FIT, fecal immunochemical test. FOBT, fecal occult blood test; HINTS, NCI Health Information Trends Survey; NCI, National Cancer Institute

This trend was observed in studies utilizing national data, Medicaid enrollment and claims data, Behavioral Risk Factor Surveillance Survey (BRFSS) data, Medicare Current Beneficiary Survey data, Electronic Medical Record data, and self-developed survey data.^26-43^ Urban individuals demonstrated significantly higher odds of reporting time-appropriate colonoscopy or flexible sigmoidoscopy.^26-30,33-35,37,41,43^ Rural populations, especially those in persistently poor or isolated rural areas, had significantly lower odds of reporting CRC screening.^28,33^ Rural individuals also exhibited lower odds of FOBT, FIT, colonoscopy, sigmoidoscopy, and overall up-to-date screening compared to their urban counterparts.^26-43^ Different definitions of rural-urban areas also consistently demonstrated higher CRC screening rates in more urban areas, irrespective of the number of categories used to define rural-urban populations.^26-43^ Rates of FOBT screening were significantly higher in rural individuals only in one study using commercial claims data (62.0% vs 38.0% urban, *P* < .0001).^43^ Rural individuals in this study had 56% higher odds of a claim for FOBT (OR = 1.56, 95% CI, 1.45-1.69), with similar odds of a claim for a colonoscopy (OR = 1.09, 95% CI, 0.98-1.14).^43^

### Studies Reporting CRC Stage at Diagnosis Disparities

A total of 282,777 individuals were evaluated across 8 studies assessing disparities in CRC stage at diagnosis (265 923 urban, 16 854 rural).^44-51^ Of the 8 studies reporting on CRC staging at diagnosis, 5 used the SEER staging classification system, one used AJCC 7th Edition Staging Manual criteria, and two did not report definitions used (Table 3).^44-51^ Half of the studies used national data (SEER Registry or North American Association of Cancer Registries data).^47,49-51^ Three studies evaluated rural-urban differences using early-stage (“in situ” or “localized”) or late-stage (“regional” or “distant”) categories, and 3 reported stage at diagnosis using numeric staging systems.^45-50^ Overall, rural individuals (especially those in isolated rural areas) showed slightly higher odds of both early- and late-stage diagnoses compared to urban individuals.^44-51^ However, there were no significant differences in staging between rural and urban populations in most studies.^44-51^ One study reported similar odds of advanced stages at diagnosis between rural and urban individuals (OR = 0.98, 95% CI, 0.81-1.19) but significantly lower odds of cancer-directed surgery among privately ensured rural individuals (OR = 0.68, 95% CI, 0.52-0.89).^45^

**Table 3.** Colorectal cancer staging study characteristics.

Author (Year)	Setting	Year(s) evaluated	Data source	Staging definition	Stage categories	Urban population (*n*, %)	Urban staging (*n*, %)	Rural population (*n*, %)	Rural staging (*n*, %)
Hines (2014)	State (GA)	2000-2007	Georgia Comprehensive Cancer Registry	SEER Staging Classification System	Late-stage, Localized, Regional, Regional + LN, Distant	17 231 (84.2%)	Late-stage: 45.2% Localized: 41.1% Regional: 13.6% Regional + LN: 25.2% Distant: 20.1%	3213 (15.7%)	Late-stage: 45.8% Localized: 39.6% Regional: 14.6% Regional + LN: 25.6% Distant: 20.2%
Leech (2022)	State (NC)	2010-2015	North Carolina Cancer Registry	AJCC 7th Edition Staging Manual	Stages 0, I, II, III, IV	3978 (75.6%)	Stages 0-III: 3035 (76.3%) Stage IV: 943 (23.7%)	1,282 (24.4%)	Stages 0-III: 972 (75.8%) Stage IV: 310 (24.2%)
Lin (2017)	State (SD)	2001-2012	South Dakota Cancer Registry	SEER Staging Classification System	Early stage, late stage^*^	1664 (34.1%)	Early stage: 765 (45.0%) Late stage: 899 (54.0%)	3 214 (65.9%)	Early stage: 1450 (45.0%) Late stage: 1764 (54.0%)
Zahnd (2018)	National	2009-2013	North American Association of Central Cancer Registries	SEER Staging Classification System	Early stage, late stage^*^	NR	Localized: 15.4 diagnoses per 100 000 Distant: 8.0 diagnoses per 100 000	NR	Localized: 16.4 diagnoses per 100 000 Distant: 9.1 diagnoses per 100 000
Risser (2012)	State (TX)	2004-2008	Texas Cancer Registry	SEER Staging Classification System	Early stage, late stage^*^	NR	NR	NR	NR
Paquette (2007)	National	2000-2003	SEER Registry	SEER Staging Classification System	Stages I, II, III, IV	114,769 (95.7%)	Stage I: 31 331 (27.3%) Stage II: 34 271 (29.1%) Stage III: 28 922 (25.2%) Stage IV: 21 232 (18.5%)	5,149 (4.3%)	Stage I: 1442 (28.0%) Stage II: 1504 (29.2%) Stage III: 1282 (24.9%) Stage IV: 922 (17.9%)
Andrilla (2020)	National	2010-2014	SEER Registry	NR	Stages I, II, III, IV (“late stage”)	128,281 (96.7%)	Stage 0: 9922 (7.7%) Stage I: 31 618 (24.6%) Stage II: 30 697 (23.9%) Stage III: 31 148 (24.3%) Stage IV: 24 882 (19.4%)	3,996 (3.0%)	Stage 0: 281 (7.0%) Stage I: 994 (24.9%) Stage II: 969 (24.3%) Stage III: 933 (23.3%) Stage IV: 849 (21.2%)
Zahnd (2018)	National	2009-2013	North American Association of Central Cancer Registries	NR	NR	NR	40.1 diagnoses per 100 000	NR	43.9 diagnoses per 100 000

^*^“Early Stage”: in situ or localized; “late stage”: regional or distant.

Abbreviations: AJCC, American Joint Committee on Cancer; LN, lymph node; NR, not reported; SEER, Surveillance, Epidemiology, and End Results Program.

### Studies Reporting CRC Treatment Disparities

Twelve studies investigated treatment disparities among a total of 6 842 538 individuals (5 700931 urban, 1 141 607 rural Table 4).^44,52-62^ Six studies assessed surgical procedures, reporting higher rates of laparoscopic colectomies in urban individuals and higher rates of open resections in rural individuals of various CRC stages (I-IV).^52,57-61^ Four studies examined differences in chemotherapy or radiation administration between rural-urban patients with CRC.^55,56,62^ An analysis of rural individuals with stage III CRC reported higher rates of no adjuvant chemotherapy, adjuvant 5-FU only, and lower rates of adjuvant oxaliplatin compared to urban individuals.^55^ Neoadjuvant chemoradiotherapy rates were similar across urban and rural areas, with CRC stage II-III rural individuals having the highest odds of treatment.^56^ Variation was observed in pre- and post-operative neoadjuvant radiation rates between urban and rural hospitals treating patients with stage I-IV CRC, with rural hospitals having a non-significant lower rate of post-operative radiation use (23.0% urban vs 3.5% rural, *P* = .08).^54^ Compliance with high-quality measures, such as lymph node removal and adjuvant chemotherapy, was lower in rural patients with stage II colon cancer and stages II-III rectal cancer.^53^ Overall, rural residents had lower odds of receiving adjuvant chemotherapy compared to urban residents.^44,56,61,62^

**Table 4.** Colorectal cancer treatment study characteristics.

Author (year)	Setting	Year(s) evaluated	Data source	CRC stages evaluated	Treatments evaluated	Urban population (*n*, %)	Urban treatment (*n*, %)	Rural population (*n*, %)	Rural treatment (*n*, %)
Al Nasser (2014)	National	2009	NIS	I, II, III, IV	Laparoscopic colectomy	14 502 (90.4%)	4337 (34.1%)	1548 (9.6%)	291 (18.8%)
Parikh-Patel (2021)	State (CA)	2004-2017	California Cancer Registry	NR	High-quality care^*^	13 075 (39.2%)	NR	20 294 (60.8%)	NR
Stewart (2013)	State (PA)	2004-2006	Pennsylvania Cancer Registry, Highmark Private Insurance Claims	I, II, III, IV	Neoadjuvant radiation	NR	62.2%	NR	57.7%
Panchal (2016)	National	2004-2009	SEER-Medicare	III	Adjuvant oxaliplatin, adjuvant 5FU	468 (71.6%)	Oxaliplatin: 145 (31.0%) 5FU: 71 (15.2%)	186 (28.4%)	Oxaliplatin: 41 (22.0%) 5FU: 37 (19.9%)
Monson (2014)	National	2006-2011	NCDB	II, III	Neoadjuvant chemoradiotherapy	5309 (87.0%)	3984 (75.0%)^*^	792 (13.0%)	607 (76.6%)^*^
Moghadamyeghaneh (2015)	National	2009-2012	NIS	NR	Laparoscopic colectomy	168 145 (87.5%)	NR	23 918 (12.5%)	NR
Patel (2022)	National	2008-2017	NIS	Malignant	Laparoscopic colectomy	174 079 (68.1%)	MIS: 106 221 (61.0%)^**^	81 688 (31.9%)	MIS: 26 939 (33.0%)^**^
Chioreso (2019)	National	2007-2011	SEER-Medicare	II, III	Rectal cancer surgical resection	1248 (78.0%)	811 (65.0%)	353 (22.0%)	155 (44.0%)
Gruber (2015)	State (NE)	2008-2011	Nebraska Cancer Registry	NR	Laparoscopic colectomy, open resection	427 (44.1%)	LR: 230 (53.9%) OR: 171 (40.1%)	541 (55.9%)	LR: 249 (46.1%) OR: 324 (59.9%)
Short (2016)	State (PA, KY, NC)	2006-2008	State Cancer Registries linked to Medicare	I, II, III	Surgical resection, LN removal	1414 (55.4%)	1004 (71.0%)	1138 (44.6%)	728 (64%)
Hao (2011)	State (GA)	2000-2004	GCCR	II (colon), II/III (rectal)	Guideline-directed adjuvant 5FU, adjuvant 5FU + leucovorin or with other cytotoxic agent^**^	5 305 033 (84.0%)	2 885 938 (54.4%)	1 008 289 (16.0%)	510 194 (50.6%)
Hines (2014)	State (GA)	2000-2007	GCCR	Any	Surgical resection + chemotherapy + radiotherapy	17 231 (84.3%)	SR: 15 429 (88.5%) Chemotherapy: 5858 (34.0%) Radiation: 2188 (12.7%)	3213 (15.7%)	SR: 2879 (89.6%) Chemotherapy: 1172 (36.5%) Radiation: 395 (12.3%)

^*^Defined using 11 performance measures established by the Commission of Cancer Quality.

^**^Oxaliplatin, capecitabine, irinotecan.

Abbreviations: 5FU, 5-fluorouracil GCCR, Georgia Comprehensive Cancer Registry; LN, lymph node; LR, laparoscopic resection; MIS, Minimally invasive surgery; NCDB, National Cancer Database; NIS, Nationwide Inpatient Sample; OR, open resection; SEER, Surveillance, Epidemiology, and End Results Program.

### Studies Reporting CRC Survivorship Care Disparities

One study assessed rural-urban disparities in CRC survivorship care (168 urban individuals, 109 rural individuals).^63^ McDougall et al evaluated adherence to surveillance colonoscopy in 30-74 year old CRC survivors at 1, 3, 5, and 10 years post-diagnosis using New Mexico Cancer Registry data.^63^ Compared to urban survivors, rural survivors were 2.28 times (95% CI, 1.07-4.85) more likely to report nonadherence to surveillance colonoscopy guidelines.^63^ Moreover, financial hardship was independently associated with nonadherence to surveillance colonoscopies (OR = 2.17, 95% CI, 1.01-4.5).^63^

### Meta-Analysis of CRC Screening Studies

**Figure 2.**
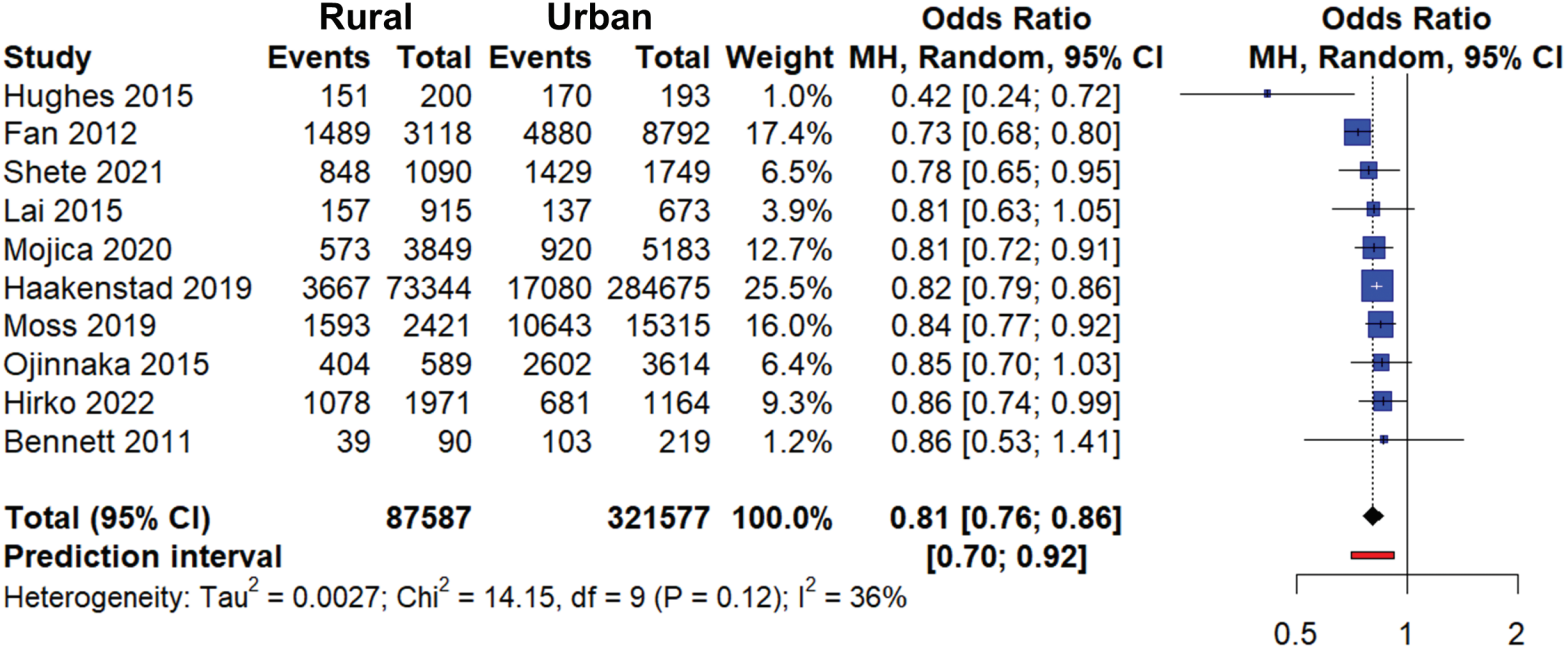
Meta-analysis forest plot, any colorectal cancer screening. Abbreviations: CI, confidence interval; MH, Mantel-Haenszel.

Results of the initial analysis of studies reporting disparities in CRC screening between urban and rural individuals may be found in Supplementary Materials.^26-36,38-40^ Due to the high heterogeneity of the initial meta-analysis (*I*^2^ = 94%, tau *P*-value > .05), 4 outliers were removed using methods described previously.^26,27,29,35^ Final results are visualized in Fig. 2. Overall, rural individuals had a 19% lower likelihood of reporting any type of CRC screening at any point in time compared to urban individuals (pooled OR = 0.81, 95% CI, 0.76-0.86). Heterogeneity was acceptable (*I*^2^ = 36%, tau 95% CI, 0.0-0.31, *Q P*-value = .1171). Funnel plots and Peter’s test revealed very little presence of small-study publication bias (Peter’s test *P*-value = .42).

## Discussion

In this analysis, we found substantial data supporting a disparity in CRC screening between urban and rural CRC individuals, minimal evidence supporting poorer standard of care treatment for rural patients with CRC, mixed evidence suggesting that rural patients with CRC had a higher stage at diagnosis, and only one study evaluating survivorship care disparities. Our results for CRC screening were consistent with previously published data. One included study directly assessed the effects of limited access to care due to cost by describing improved CRC screening rates after introduction of the Affordable Care Act (ACA), which improved access to CRC screening by eliminating financial barriers.^40^ However, the persistence of this gap in screening over time suggests that non-financial factors may still exist to limit rural utilization of appropriate CRC screening. Our mixed results with respect to disparities in stage at diagnosis largely differ from other studies evaluating this rural-urban disparity for other types of cancers.^47,65,66^ This may have been due to a variety of reasons, but given the variation in time periods assessed among the included studies evaluating the CRC stage at diagnosis, we note that it is challenging to account for the long latency period between environmental exposures (rural-urban status) and cancer diagnosis. Furthermore, we did not include a specific definition of “stage” at diagnosis for this review, leading to the inclusion of studies with various staging definitions used which may have contributed to our mixed results. We found, paradoxically, that research to date suggests a disparity among rural and urban patients with respect to CRC screening, but the distribution of stages at diagnosis was very similar between the 2. While there may be several explanations for this observation, one hypothesis stems from a large amount of missing staging/registry data for patients who tend to do very poorly, such as rural patients with cancer.^67^ This may have biased results in staging studies more favorably for rural patients with CRC. Our results indicating a treatment disparity are present is similar to studies for other types of cancer, suggesting that barriers may exist in access to medical or surgical oncology services for rural patients.^68-71^ Finally, the single study evaluating CRC survivorship care differences noted a disparity in access to and adherence to survivorship CRC surveillance care.^63^ As patients with CRC live longer lives, it is critical to understand and measure how disparities noted upstream in the cancer care continuum may carry over to survivorship care and affect clinical outcomes. Given the lack of data in this area, researchers may want to focus more on disparities of the quality of survivorship care received between rural and urban CRC survivors.

Underlying mechanisms driving the rural-urban disparities we observed in this study may be better understood through a framework that considers factors such as low socioeconomic status, lack of insurance coverage, limited healthcare access, and transportation issues.^14^ These factors in turn contribute toward inadequate surveillance, late-stage diagnosis, and lower quality of cancer treatment.^14^ Qualitative research has expanded on some of these underlying mechanisms. For example, work from Lee et al describes distance to travel in rural areas as a consistently reported barrier toward achieving appropriate CRC screening.^72^ Others have reported clear barriers for rural patients in receiving timely specialist care after diagnosis, and a lack of clear communication between providers and patients, which may lead to the poorer care received that we observed in this analysis.^73,74^ A series of structured interviews among rural Nebraskan patients with CRC also suggests that providers may not even discuss alternative screening methods unless rural patients with CRC resist colonoscopies.^75^ Ultimately, the multitude of rural barriers to appropriate receipt of CRC screening, early stage at diagnosis, standard of care treatment, and survivorship care are complex and interdependent and involve patients, providers, and community-level effects. Therefore, a multifactorial and cross-functional effort between patients, providers, local decision makers, and national policymakers is essential for addressing these underlying mechanisms. For example, national and state-level policymakers may advocate for the expansion of CRC screening and treatment services covered under Medicaid, which in turn may increase the timely use of screening and treatment for patients with lower socioeconomic status. These policymakers may also opt to increase financial support for local rural providers to coordinate with other urban cancer centers, such as through the National Cancer Institute Community Oncology Research Program. Finally, national funding bodies may consider pushing to incentivize rural recruitment into CRC-related clinical trials, potentially improving access to timely care.

A notable trend observed in this review was the wide variety of used rural-urban definitions. This definition variability severely limited our synthesis of results. While federal bodies such as the National Institutes of Health (NIH) have often called for harmonization of person-level and contextual variables for research purposes, there is currently no call for standardization of the rural-urban definition. While this may be for several reasons, we recognize the difficulty in utilizing only one standard definition for rural-urban status. For example, certain definitions, such as those used by the US Census Bureau or the OMB, focus exclusively on population density as a means of defining rural-urban categories, while others use more specific criteria that incorporate other contextual factors, such as proximity to urban areas or commuting distance.^76,77^ Moreover, certain definitions are confined to the US census-tract level, which may change over time and are large enough such that they may contain both “rural” and “urban” areas. Others utilize county-level estimates (eg, OMB definition, RUCC codes).^77,78^ Finally, certain taxonomies can further subdivide areas past rural or urban status, which may improve the precision of certain estimates, but comes with the risk of inadequate sample size. We note that the use of varying definitions of rural-urban status is also frequently a product of data availability. For example, SEER data, curated by the National Cancer Institute (NCI), only includes spatial data using RUCA codes, RUCC codes, or MSAs. RUCA and RUCC codes, in particular, were fairly prominent in our review.^79^ This may be because RUCA and RUCC codes combine population density, proximity to urban areas, and other factors.^78,80^ While RUCA codes are measured at the census tract level, RUCC codes are smaller subdivisions of county areas.^78,80^ Because county areas are smaller than census tracts, differences in results may be observed when comparing studies that use these different definitions. Due to these nuances in how one may define “rural-urban” status, there naturally exists disagreement as to which definition is better suited for analysis. The consequences of a lack of consensus surrounding best practices for rural-urban criteria are evident when attempting to synthesize published data, as various definitions can introduce bias. For example, Hao et al and Hines et al reported similar rates of therapy between urban and rural patients with CRC, while Panchal et al reported much lower rates among rural individuals. While there may be several reasons for this observation, it is worth noting that Hao et al and Hines et al used the RUCA criteria for rural-urban status, which allowed for the inclusion of smaller towns with local commuting to urban areas as “rural.” In contrast, Panchal et al used a stricter definition of rural using RUCC codes, limiting “rural” to areas with <2500 residents. Analyses utilizing rural-urban criteria that allow for urban-adjacent areas to qualify as “rural” may bias results toward a null hypothesis. Therefore, careful attention must also be paid to studies that report no significant difference between rural and urban receipt of CRC-related care to ensure that bias from the use of various rural-urban definitions is limited.

Outside of the challenges involving rural-urban definition, analytic considerations may also pose a challenge in the analysis of rural-urban CRC disparities. Most studies in our review used regression methods where rural status was considered as a person-level variable, instead of a contextual one. A few studies used more advanced 2-level hierarchical models where the rural-urban definition was used as a nesting level, allowing investigators to account for the effect of either the same residential or hospital location among different patients CRC. While this is a reasonable approach, one may also consider the use of cross-classified multilevel models to simultaneously account for nesting in both residential areas and hospital care areas.^81^ For example, patients with CRC may be nested within the same geographic location, but seek care in different locations. Cross-classified multilevel models provide a unique method to model the diverse settings that may affect CRC care and outcomes, yet are underused in spatial research.^81^ Alternatively, investigators may also consider the use of 2-step floating catchment area (2SFCA) methods to aid in the determination of access to healthcare services.^82^ The 2SFCA method is particularly insightful, as it not only considers the distance to care but also how accessible a provider is to a patient given the surrounding population density.^82^ For example, 2SFCA methods have been used previously to determine spatial access to substance use disorder treatment services, accessibility to ICU beds during the COVID-19 pandemic, and access to primary care providers.^83-85^ Overall, while a variety of methods exist to quantify spatial disparities in CRC screening and care, future investigators may want to consider the use of different, more robust methods of analysis.

This analysis had several limitations to acknowledge, such as the heterogeneity of the studies included and assessed with regard to rural-urban definition, definition of “screening,” definition of “treatment,” and definition of “staging.” While we were able to perform a meta-analysis of studies with adequate statistical heterogeneity, the studies included vary widely in study design, cohort definition, and outcome definitions. Therefore, readers should cautiously interpret our meta-analysis results. Similarly, while statistically we demonstrated low publication bias for CRC screening studies, the same could not be said for studies evaluating the CRC stage at diagnosis or treatment. We also note that differences in rural-urban receipt of CRC treatment noted for this study may have been biased by each patient’s health status at baseline. For example, rural individuals may be “sicker” at baseline, leading to a difference in the type of CRC treatment received as opposed to urban individuals. We only found one study evaluating CRC survivorship care, limiting the applicability of our findings in this area. The studies included in this review were all retrospective, as no prospective studies were found. While retrospective studies confer specific strengths, an amount of selection bias is inherent to these studies, limiting results. Finally, as this was primarily a descriptive analysis in scope, we were unable to account for the effect of time on our results, which may be relevant as the studies included in this review varied widely. While it does not appear that the time period evaluated had a relationship to the disparities we noted here, important interventions such as policy (eg, Affordable Care Act) and changes in guidelines may have affected the results noted in screening, stage at diagnosis, and treatment.

## Conclusions

Our findings suggest the presence of rural disparities in equitable access to CRC screening. Investigators should focus on understanding the driving factors behind our noted disparities in CRC screening, such as inequity in socioeconomic status, distance and access to appropriate care, and appropriate healthcare coverage. Given the mixed findings for rural disparities in CRC staging at diagnosis and treatment, it may be that the largest barrier rural individuals face may simply be at the initial screening level. Decision-makers should focus their policy efforts on improving this initial access to care for rural individuals. Future investigators should also weigh the benefits and limitations of using certain definitions for rurality and may want to consider the use of more robust methods suited for spatial analysis to better understand these disparities.

## Supplementary Material

Supplementary material is available at *The Oncologist* online.

oyad347_suppl_Supplementary_Figures_S1-S3

## Data Availability

Code and data used for the meta-analysis of this study may be found at: https://tinyurl.com/3kknzasx.
