# Supplementary material for: Rural-Urban Disparities in Colorectal Cancer Screening, Diagnosis, Treatment, and Survivorship Care: A Systematic Review and Meta-Analysis
Source: Oncologist. 2024 Jan 19;29(4):e431–46. doi: 10.1093/oncolo/oyad347 (PMC10994268; doi:10.1093/oncolo/oyad347)
Supplement: oyad347_suppl_Supplementary_Figures_S1-S3 [file oyad347_suppl_supplementary_figures_s1-s3.docx]

**Supplementary Materials**

**Table S1. Systematic Literature Review Search Strategies**

| PubMed/MEDLINE | ("rural population"[MeSH Terms] OR "rural health services"[MeSH Terms] OR "rural areas"[All Fields] OR "rural communities"[All Fields] OR "rural populat*"[Title/Abstract] OR "isolated populat*"[All Fields]) AND ("suburban areas"[All Fields] OR "suburban population"[MeSH Terms] OR "suburban populat*"[Title/Abstract] OR "urban population"[MeSH Terms] OR "urban areas"[All Fields] OR "urban populat*"[Title/Abstract] OR "urban health services"[MeSH Terms]) AND ("colorectal neoplasms/diagnosis"[MeSH Terms] OR "colorectal neoplasms/epidemiology"[MeSH Terms] OR "colorectal neoplasms/etiology"[MeSH Terms] OR "Early Detection of Cancer"[MeSH Terms] OR "Disease Progression"[MeSH Terms] OR "colorectal neoplasms/rehabilitation"[MeSH Terms] OR "colorectal neoplasms/therapy"[MeSH Terms] OR "colonic neoplasms/rehabilitation"[MeSH Terms] OR "colonic neoplasms/therapy"[MeSH Terms] OR "rectal neoplasms/rehabilitation"[MeSH Terms] OR "rectal neoplasms/etiology"[MeSH Terms] OR "rectal neoplasms/epidemiology"[MeSH Terms] OR "rectal neoplasms/therapy"[MeSH Terms] OR "colonic neoplasms/epidemiology"[MeSH Terms] OR "colonic neoplasms/etiology"[MeSH Terms] OR "colon cancer"[Text Word] OR "rectal cancer"[Text Word] OR "colorectal cancer"[Text Word] OR "CRC"[Text Word]) AND "united states"[MeSH Terms] |
| --- | --- |
| EMBASE | 'rural population'/exp OR 'rural health care'/exp OR 'rural areas' OR 'rural communities' OR 'rural populat*’:ti,ab OR 'isolated populat*'  AND  ‘suburban areas’ OR 'suburban population'/exp OR ‘suburban populat*’:ti,ab OR 'urban population'/exp OR ‘urban areas’ OR ‘urban populat*’:ti,ab OR 'health service'/exp  AND  'colorectal tumor'/exp/dm_di,dm_ep,dm_et,dm_rh,dm_th OR 'colon tumor'/exp/dm_ep,dm_et,dm_rh,dm_th OR 'rectum tumor'/exp/dm_ep,dm_et,dm_rh,dm_th OR 'early cancer diagnosis'/exp OR 'disease exacerbation'/exp OR ‘colon cancer’ OR ‘rectal cancer’ OR ‘colorectal cancer’ OR ‘CRC’  AND  'United States'/exp |

**Table S2. Study Inclusion and Exclusion Criteria**

| Inclusion Criteria | - Quantitative or observational studies - Randomized controlled trials - English language - Patients diagnosed with CRC at any stage - Results reported stratified by rural/urban individuals as either a primary result or secondary - Primary aim of the study is to evaluate disparities in: screening (pre-diagnosis), stage at diagnosis, treatment of CRC, or survivorship care |
| --- | --- |
| Exclusion Criteria | - No comparative data reported between rural/urban individuals - Other systematic reviews, letters to the editor, ethnographic studies, or case studies - Data published prior to 2000 - Studies that did not report results on specific screening methods, stages at diagnosis, or types of CRC treatment |

**Table S3. PICOT Criteria**

| Criteria | Determinants |
| --- | --- |
| Population | - Participants over 18 years of age - Categorized as either rural or non-rural |
| Intervention | Screening Interventions:   - “Any screening” (no limitations) - Colonoscopy - Flexible Sigmoidoscopy - FOBT and/or FIT - Barium Enema Exam - Any other specified type of valid exam   Treatment Interventions:   - Surgical Resections - Chemotherapy (described generally, or using specific drugs) - Radiotherapy   Survivorship Care Interventions:   - Surveillance colonoscopy - Regular provider visits as described using NCCN guidelines |
| Comparator | - Rural vs non-Rural categorizations (no limitation on type of definition used) |
| Outcomes | - Percent of uptake of CRC screening - Percent/distribution of stages at diagnosis - Percent of CRC treatment type uptake - Percent of CRC survivorship care uptake |
| Time Horizon | - Data analyzed from 2000 to present |

**Figure S1. Distribution of Included State-Based Studies**


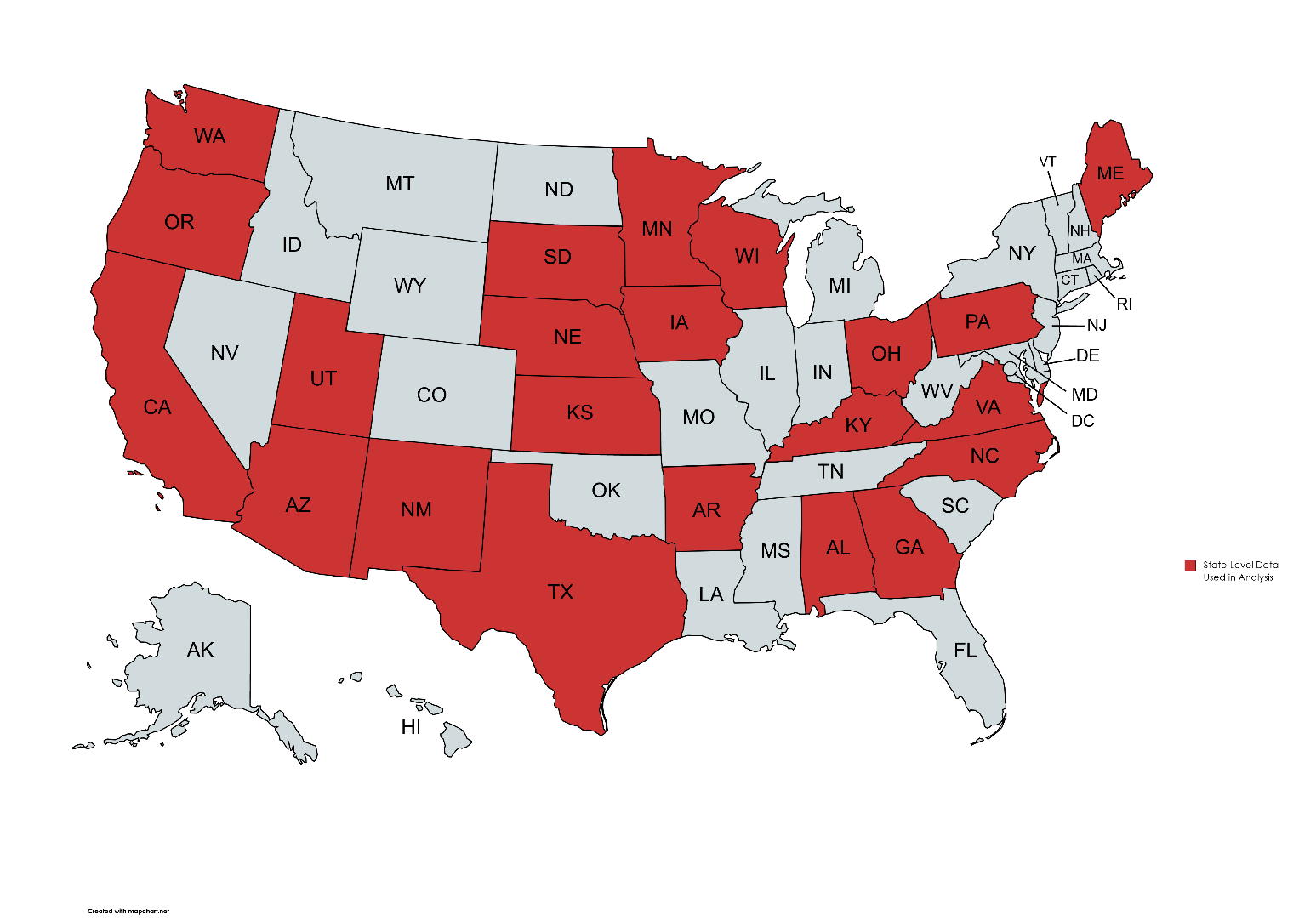


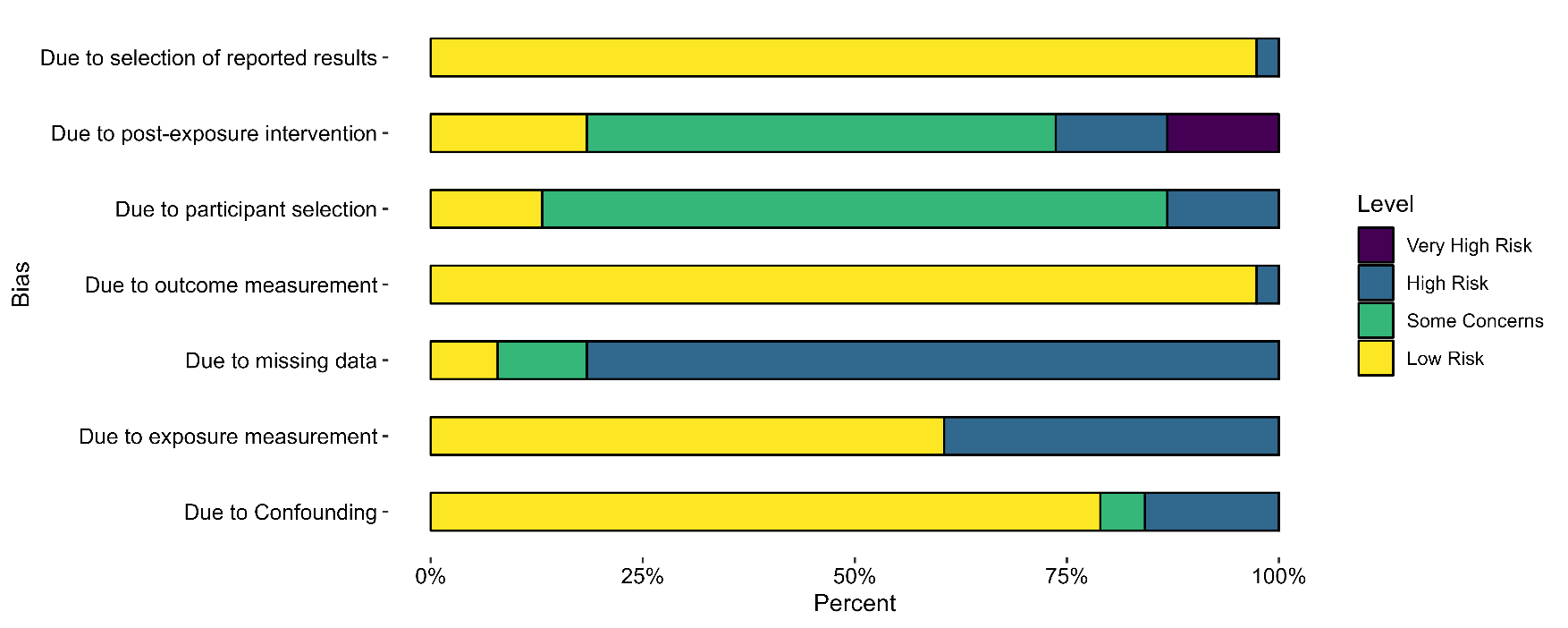
**Figure S2. Collective Risk of Bias Graph using the ROBINS-E Tool**


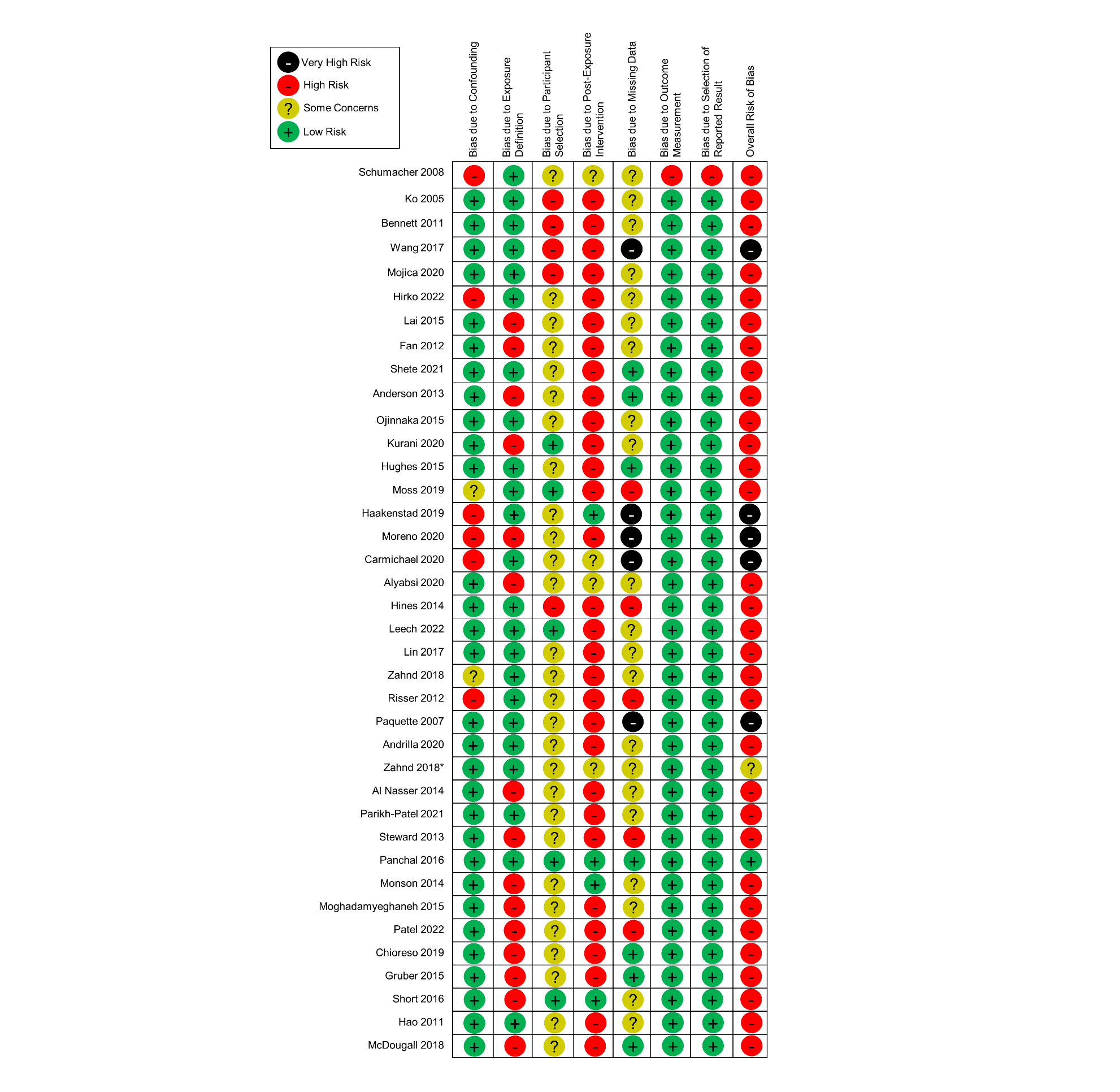
**Figure S3. Individual Study Risk of Bias Graph using the ROBINS-E Tool**

**

Figure S4. Meta-Analysis Forest Plot, Any Screening, with Outliers Included**

*Where “Events” represents Rural individuals and “Control” represents Urban individuals



**Figure S5. Contour-Enhanced Funnel Plot, Meta-Analysis of Colorectal Cancer Screening, without Outliers**
